# Identification, Characterization, and Diel Pattern of Expression of Canonical Clock Genes in *Nephrops norvegicus* (Crustacea: Decapoda) Eyestalk

**DOI:** 10.1371/journal.pone.0141893

**Published:** 2015-11-02

**Authors:** Valerio Sbragaglia, Francesco Lamanna, Audrey M. Mat, Guiomar Rotllant, Silvia Joly, Valerio Ketmaier, Horacio O. de la Iglesia, Jacopo Aguzzi

**Affiliations:** 1 Marine Science Institute, (ICM-CSIC), Barcelona, Spain; 2 Unit of Evolutionary Biology/Systematic Zoology, Institute of Biochemistry and Biology, University of Potsdam, Potsdam, Germany; 3 Department of Biology, University of Washington, Seattle, WA, United States of America; 4 Department of Biology and Biotechnology “Charles Darwin”, University of Rome “La Sapienza”, Rome, Italy; University of Ferrara, ITALY

## Abstract

The Norway lobster, *Nephrops norvegicus*, is a burrowing decapod with a rhythmic burrow emergence (24 h) governed by the circadian system. It is an important resource for European fisheries and its behavior deeply affects its availability. The current knowledge of *Nephrops* circadian biology is phenomenological as it is currently the case for almost all crustaceans. In attempt to elucidate the putative molecular mechanisms underlying circadian gene regulation in *Nephrops*, we used a transcriptomics approach on cDNA extracted from the eyestalk, a structure playing a crucial role in controlling behavior of decapods. We studied 14 male lobsters under 12–12 light-darkness blue light cycle. We used the Hiseq 2000 Illumina platform to sequence two eyestalk libraries (under light and darkness conditions) obtaining about 90 millions 100-bp paired-end reads. Trinity was used for the *de novo* reconstruction of transcriptomes; the size at which half of all assembled bases reside in contigs (N50) was equal to 1796 (light) and 2055 (darkness). We found a list of candidate clock genes and focused our attention on canonical ones: *timeless*, *period*, *clock* and *bmal1*. The cloning of assembled fragments validated Trinity outputs. The putative *Nephrops* clock genes showed high levels of identity (blastx on NCBI) with known crustacean clock gene homologs such as *Eurydice pulchra* (*period*: 47%, *timeless*: 59%, *bmal1*: 79%) and *Macrobrachium rosenbergii (clock*: 100%*)*. We also found a vertebrate-like *cryptochrome 2*. RT-qPCR showed that only *timeless* had a robust diel pattern of expression. Our data are in accordance with the current knowledge of the crustacean circadian clock, reinforcing the idea that the molecular clockwork of this group shows some differences with the established model in *Drosophila melanogaster*.

## Introduction

The Norway lobster, *Nephrops norvegicus* (hereafter referred as to *Nephrops*), is a burrowing decapod distributed in the European Atlantic Ocean and in the Mediterranean Sea, from 10 to 800 m depth [1]. It is one of the most important resources for European fisheries with landed tons of about 70000 per year (http://www.fao.org). Stock assessment and fishery management are influenced by its rhythmic pattern of burrow emergence, promoting research on its circadian regulation both in the field and in the laboratory. Emergence in the laboratory can be considered as a proxy of availability of lobsters in the wild. As a matter of fact, animals can be captured by trawling only when they are out of the burrow [2, 3] and the knowledge of mechanisms behind that behavior could be important for stock assessment of the species. Multiple aspects of biology and ecology of the Norway lobster have been investigated in the last four decades [1, 4–8]. However, molecular mechanisms governing the rhythmic behavior are not known.

The current knowledge of *Nephrops* circadian biology (and of crustaceans in general) is merely phenomenological, with very few insights on the molecular mechanisms regulating this behavior [9–11]. The molecular architecture of the circadian system in decapod crustaceans is indeed poorly known [12, 13] when compared to what has been achieved so far in other arthropods such as the fruitfly *Drosophila melanogaster* [14]. In crustacean decapods, the eyestalks and their optic ganglia play a crucial role in the modulation of neuroendocrine and behavioral rhythms. They are an important source of neuropeptides including red pigment concentrating hormone, crustacean hyperglycemic hormone, pigment dispersing hormone, typically released by X-organ sinus gland complex, as well as of small molecules, such as serotonin and melatonin, both involved in circadian regulation [15–20]. Hence, the eyestalks are a good candidate for the search of genes involved in circadian regulation (clock genes) and their temporal pattern of expression.

The striking level of conservation of the molecular architecture of the circadian system among eukaryotes implies that putative clock genes of *Nephrops* could show homology with those of the phylogenetically closest arthropod model organism such as the fruitfly *Drosophila melanogaster* [21]. On the other hand, the advent of next-generation sequencing (NGS) technologies allows delivering new, fast, and accurate information of wide portions of organisms’ genomes, providing large number of reads in non-model species in which previous genomic information is unavailable [22, 23]. Recent advances in assembly algorithms allow using NGS technologies that produce short reads; computational time in reads assembly is reduced by using a paired-end protocol without decreasing accuracy [24, 25]. RNA-sequencing (RNA-seq) is a method that uses NGS to gain information on transcriptomes (the transcribed portion of the genome). The fact that RNA-seq is not based on a hybridization-based approach using preexisting sequences (e.g. microarrays) makes this technique less biased and very attractive for non-model species such as the Norway lobster, where reference genomic data are not available.

In the present study, we sequenced and *de novo* assembled the *Nephrops norvegicus* eyestalk transcriptome. Because of the paramount role that clock genes likely have in regulating the rhythmicity of burrow emergence behavior, we annotated canonical clock gene homologs (e.g. *period*, *timeless*, *clock*, *bmal1*) and assessed their diel pattern of expression (RT-qPCR) in relation to the burrow emergence rhythms.

## Materials and Methods

### Behavioral analysis

#### Sampling and housing

Sampling and laboratory experiments followed the local legislation regarding animal’s welfare. Animal sampling was conducted with the permission of the local authority (Generalitat de Catalunya). The species used in this study is not an endangered or protected species.

Lobsters were collected exclusively at nighttime by a commercial trawler on the coastal shelf area (100 m) off the Ebro delta (Tarragona, Spain). Operations on the deck and the transportation of lobsters to the laboratory occurred at night and all deck operations were performed under dim red light, following the methods described by Aguzzi and colleagues [26]. In the laboratory, animals were acclimated within a light-proof isolated chamber: constant temperature (13±1°C), random feeding time, blue monochromatic light-dark (LD) cycle matching the natural one. More details on the acclimation are described by Sbragaglia and colleagues [27].

Burrow emergence rhythm was tracked in the laboratory using an actograph under monochromatic blue light (472 nm) and equipped with an artificial burrow. Automated video image analysis quantified animal displacements out of their burrows, for further details see Sbragaglia and colleagues [28]. The 14 individuals used in this study were adult intermoult *Nephrops* males (CL = 44.24±5.25 mm). Animals were acclimated for at least 40 days. Behavioral tests were carried out as follow: 12h-12h LD cycle (lights-ON at 08:00 h and lights-OFF at 20:00 h), with an intensity during light hours of 4·10^−3^ μE/m^2^/s, simulating light intensity at depth of about 150 m [29]. During darkness hours video recording was accomplished using infrared (850 nm) light. Blue lights-ON and -OFF were progressively attained and extinguished within 30 min, in order to avoid photoreceptor degeneration (i.e. rhabdomere deterioration and visual pigments photolysis), which occurs when animals are exposed to sudden bright light exposure [30]. All trials were conducted under constant temperature (13±1°C) for 10 days. Eyestalks were dissected during the last day of the experiment. Sampling and laboratory experiments followed the local legislation regarding animal’s welfare.

#### Behavioral data analysis

Behavioral analyses were performed using the software *Eltemps* (www.el-temps.com). Chi-square periodogram [31] was used to scan for the presence of significant (*p* < 0.05) periodicity in the range 10–28 h and percentage of variance (%V) explained by each period is reported as a measure of rhythms' robustness [32]. Waveform analysis (24-h based) was carried out in order to identify the behavioral phenotype (nocturnal or diurnal) and the “midline estimating statistic of rhythm” (MESOR) was also computed. The percentage of the activity (area under the waveform curve) during darkness was calculated to determine the nocturnal or diurnal phenotype of the lobsters. Lobsters were considered nocturnal when more than 60% of locomotor activity was concentrated during darkness.

### Transcriptome analysis

#### Eyestalk dissection and RNA extraction

Lobsters were anesthetized on ice for 15 minutes and their eyestalks dissected at the middle of the photophase (n = 4) and at the middle of the scotophase (n = 4). The cuticle and the retina were rapidly eliminated using a stereoscope under dim red light and the remaining tissue immediately transferred to RNA-later tissue collection (Invitrogen Inc.) and stored at -80°. Total RNA isolation was performed using the column-based RNeasy^®^ Mini Kit (Qiagen Inc.) following manufacturer instructions.

#### Sequencing and quality check

Before sequencing, the quality of the RNA integrity was checked using Agilent Technologies 2100 Bioanalyzer. Two samples (one for the photophase: NEP-L; and the other for the scotophase: NEP-D) were chosen for the construction of non-normalized cDNA libraries. The mRNA fraction of the total RNA was converted into a library of template molecules suitable for subsequent cluster generation using the reagents provided in the Illumina TruSeq RNA Sample Preparation Kit. Sequencing was performed using one channel of a HiSeq 2000 Illumina Sequencing System (paired-end, 100bp). FastQC (v0.10.0) was used to provide the quality control checks on raw sequence data coming from high throughput sequencing pipelines (http://www.bioinformatics.babraham.ac.uk).

#### Data analysis

Trinity r2011-11-26 (http://trinityrnaseq.sourceforge.net) was used for the *de novo* reconstruction of transcriptomes from the read data [33, 34]. Transcriptomes were first assembled separately (NEP-L and NEP-D) and then together (NEP-comb). In order to get the species distribution of the annotated hits of transcripts the combined transcriptome (NEP-Comb) was blasted against the Uniprot database (http://www.uniprot.org) using a stand-alone version of the blastX tool (v20120420) and setting the E-value cutoff to 10^−6^. BlastX translates the query sequence in all six possible reading frames and provides combined significance statistics for hits to different frames (http://blast.ncbi.nlm.nih.gov). Then, to assign putative gene functions, contigs from NEP-L and NEP-D were blasted again. Estimates of the numbers of annotated contigs that matched to known genes from the NCBI non-redundant protein sequence database were made and functional categories of the predicted genes were obtained by extracting the relative Gene Ontology (GO) terms from the blastX output (http://www.geneontology.org) [35]. The grouped sets of GO terms was then subjected to a Fisher's exact test, using False Discovery Rate (FDR) *p*-value correction for multiple comparisons (*p* < 0.05), in order to find under- or over-represented terms between the two transcriptomes.

In order to find transcripts in our dataset that could be considered as putative clock genes or genes related to the circadian system, we screened the description of the annotated sequences looking for the following key terms: "circadian", "rhythmic", "entrainment".

The RSEM (v1.2.3) software tool [36] was used for quantifying transcript abundances from RNA-seq data. The assembly of the transcriptome derived from the sample NEP-D was used as a reference in order to obtain comparable expression levels between the two samples. Statistical significance in transcript abundance differences across the two transcriptomes was assessed using the R package EBSeq v1.1.5 [37].

### Cloning and quantitative real-time RT-PCR

#### RNA extraction and cDNA synthesis

The eyestalks were dissected in 12 lobsters at four different time points (three individuals at each time point): 07:30 (just before lights-ON), 13:30 (at the middle of photophase), 19:30 (just before lights-OFF), and 01:30 (at the middle of scotophase). The cuticle and the retina were rapidly eliminated using a stereoscope under dim red light and the remaining tissue immediately frozen in liquid nitrogen and then stored at -80°. Eyestalk tissue was homogenized with 0.5 mL of Trizol and total RNA was extracted with chloroform, precipitated with isopropanol and washed with 75% ethanol. Pellets were suspended in 25 μL DEPC-water and stored at -80°C. The quality of RNA was checked on gel electrophoresis and by absorbance ratio (A260/A280 nm) > 1.8. No clear signs of DNA contamination were found so we decided against a DNase treatment. Concentration was assessed by absorbance at 260 nm, using a ND-1000 spectrophotometer (NanoDrop Technologies). One μg of total RNA was reverse transcribed into cDNA using Superscript III (Invitrogen) and random hexamers following manufacturer’s instructions.

#### Cloning and sequencing

We used PCR amplification to clone the putative *Nephrops* clock genes previously identified by blasting the *de novo* assembled transcriptome in Uniprot. This step also allowed assessing the fidelity of the assembling provided by trinity. We selected the contigs that matched with canonical clock genes *timeless*, *period*, *clock* and *bmal1* in order to study their expression using quantitative RT-qPCR. We retrieved a cDNA sequence of *Homarus americanus* (accession AF399872) encoding *α-actin* as housekeeping gene and *18S* (retrieved from the assemble transcriptome of *Nephrops*) was added as second housekeeping gene. Primers used for cloning were designed using MacVector 11.1.2 (for details see Table A in S1 Text). cDNAs for cloning and sequencing were obtained from a pool of eyestalks dissected during light and darkness conditions following the protocol described above.

The PCR protocol consisted of one denaturating step at 94°C for 2 min followed by 30 to 35 cycles each consisting of 94°C for 30 s, annealing temperature (see Table A in S1 Text) for 30 s and 72°C for 40 to 100 s (60 s per kb). The PCR product was then cloned using the StrataClone PCR cloning kit (Agilent Technologies, USA) following manufacturer’s instructions. Plasmids were then purified using the PureLink Quick Plasmid Miniprep Kits (Invitrogen, USA) and sequenced for both strands using the T3 and T7 universal primers (Genewiz, Inc., USA).

Alignments between assembled and cloned sequences (as well as *α-actin* from *H*. *americanus* and *N*. *norvegicus*) were performed using EMBOSS Water open software suit (CluscalW2, http://www.ebi.ac.uk). Blastx was used to compare the translated protein products of the contigs against NCBI database. SMART was used to identify conserved domains and structural motifs of protein (http://smart.embl-heidelberg.de; [38,39]), then amino acids sequences were blasted against NCBI data base directly from SMART (http://blast.ncbi.nlm.nih.gov/Blast.cgi; [40]).

#### RT-qPCR

Following Bustin and colleagues [41] the great part of the fundamentals information regarding the RT-qPCR experiment is included (with integrations in the supporting information). RT-qPCR reactions were carried out using an ABI 7900 HT (Applied Biosystems). Primers for *timeless*, *period*, *bmal1*, *clock*, *α-actin and 18S* (these last two as endogenous reference gene to standardize the expression levels) were designed using MacVector on the cloned sequences (Table B in S1 Text). Primers were tested before using a RT-PCR touch-down protocol with the following settings: 94°C for 5 min, 10 cycles (94°C for 30 s, 55°C + 0.5°C each cycle for 30 s, 72°C for 30 s), 30 cycles (94°C for 30 s, 55°C for 30 s, 72°C for 30 s), 72°C for 7 min. Primers amplification efficiencies were tested by linear regression analysis from a cDNA dilution series and by running a melting curve (95°C for 15 s, 60°C for 15 s and 95°C for 15 s). Efficiency (E = 10(-1/slope)), showed values between 1.9 and 2.3, standard curves ranging from –2.5 to –3.6 and linear correlations (R^2^) higher than 0.97 were recorded. cDNA was diluted 1:10 for all genes.

Cycling conditions of the RT-qPCR were: decontamination step (50°C for 2 min), activation step (95°C for 10 min), 40 cycles of denaturation (95°C for 15 s) and one annealing/extension step (60°C for 1 min). A final dissociation step was also added (95°C for 15 s and 60°C for 15 s). Each sample was run in triplicate in 384-well plates. The reaction volume (10 μL) was composed by 2 μL of 5x PyroTaq EvaGreen qPCR Mix Plus, ROX (Cultek Molecular Bioline), 6 μL distilled water, 1 μL primer mix at a 10 mM concentration and 1 μL of cDNA. Duplicate negative controls were also run. SDS 2.3 software (Applied Biosystems) was used to collect raw data. The transcript levels of the target genes were normalized to the reference genes *α-actin and 18S* and fold change was calculated following the 2^ΔΔCT^ method [42]. We decided to use an *actin* as a reference gene for the expression stability of this family of proteins, similarly to what already done with *β-actin* gene in the Antarctic Krill (*Euphausia superba*) [43]. We also used the gene *18S* because it was previously validated as a control gene with time course data in *E*. *superba* [44]. The fold change was calculated using one of the sampling points (07:30) as a control or calibrator (more details regarding the calculation and propagation of errors are described in Appendix A and B of S1 Text).

Statistical analysis was performed using the 2Δ*CT* values (see Appendix C in S1 Text for more details on the calculation). Results from 2Δ*CT* calculation were then checked for normality (Shapiro-Wilkoxon test), homoscedasticity of variance (Levene’s mean test) and a one-way ANOVA test was used to assess differences among sampling times using the Sigma Plot (12.5) software.

## Results

### Behavioral analysis

**Fig 1:**
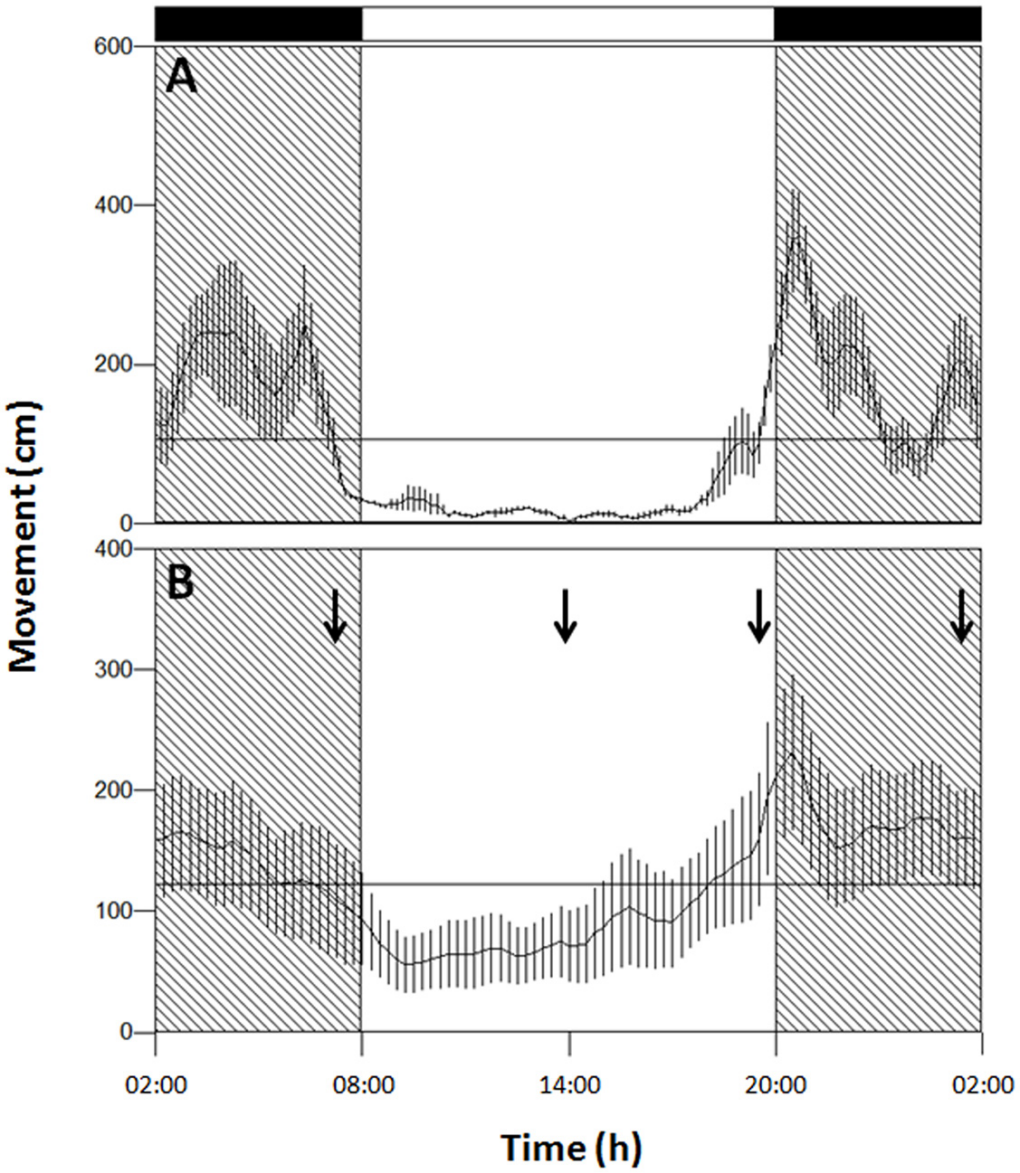
Waveform analysis. Waveform (24 h) analysis throughout the 10 days of experiment for a representative lobster (**A**) and averaged for the 14 lobsters used during the study (**B**). Activity is reported as displacement (cm) out of the burrow. Black and white bars represent darkness and light hours, respectively. Shadowed areas represent scotophase. Vertical lines represent the standard error of the mean and the horizontal line represents the MESOR. Arrows in **B** stay for the sampling points at which eyestalk were dissected for the RT-qPCR experiment.

Fig 1A shows the analysis of behavioral activity rhythms for one representative lobster used in the study. The Chi-square periodogram analysis identified a significant periodicity in burrow emergence rhythms of all animals, mean±SEM: 24.09±0.12h (37.37±4.68%V). Waveform analysis showed a nocturnal burrow emergence activity for all lobsters (mean±SEM: 71.27±3.35% of locomotor activity during darkness). The average waveform for all 14 individuals (Fig 1B) revealed an anticipatory peak of activity just before lights-OFF, which was also evident in the waveform for each individual (e.g. Fig 1A). Row Locomotor activity data are available in the Appendix A of the S1 File.

### Transcriptome analysis

**Fig 2:**
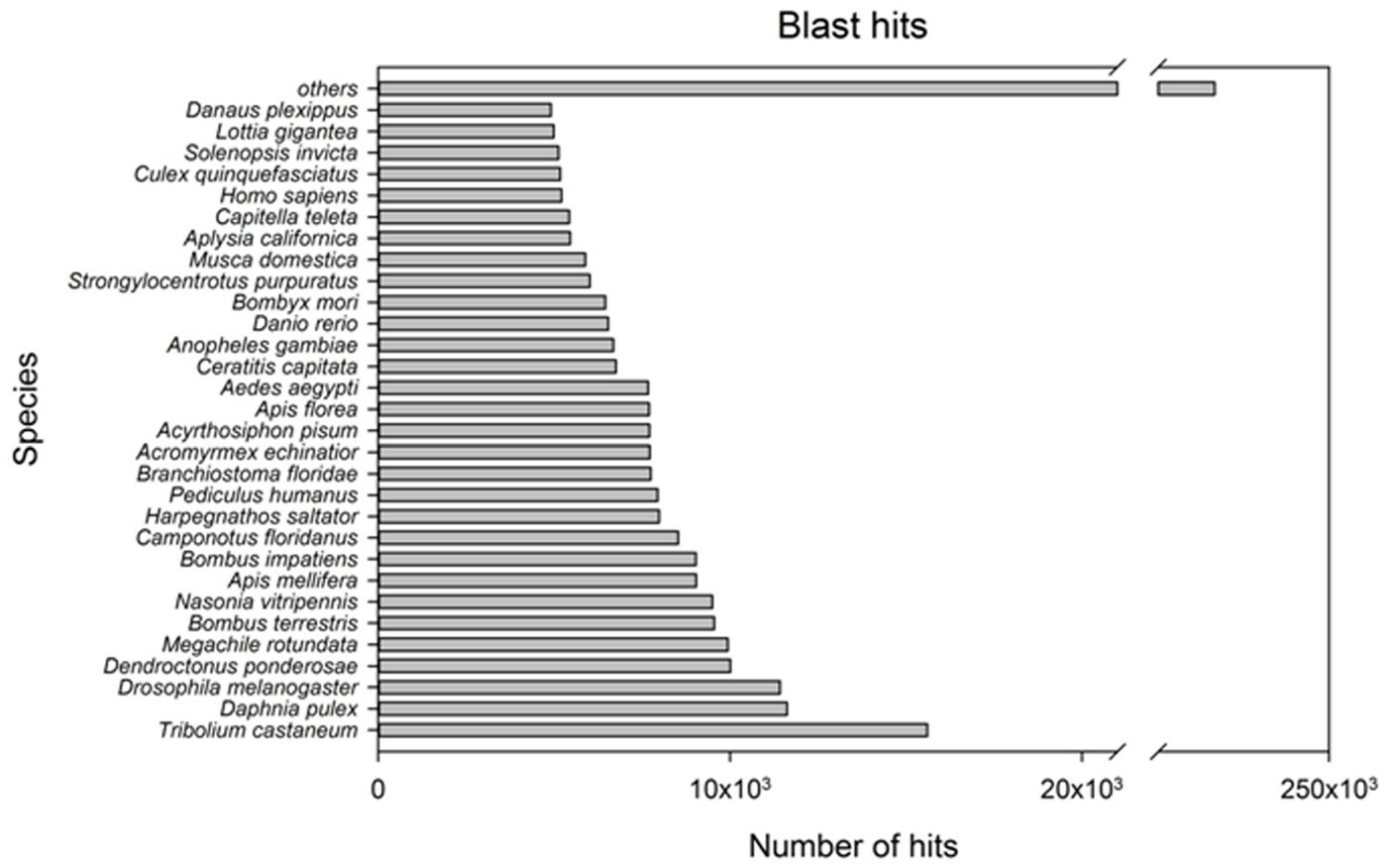
Species distribution of annotated transcripts of the merged transcriptome (NEP-comb). The species distribution of the annotated hits of transcripts against the NCBI non-redundant protein sequence database (E-value cutoff to 10^−6^). Horizontal bars depict the number of hits for each one of the species. Only 30 species in order of number of annotated hits were presented, while the hits of all the other species are summed into the bar “*others”*.

**Fig 3:**
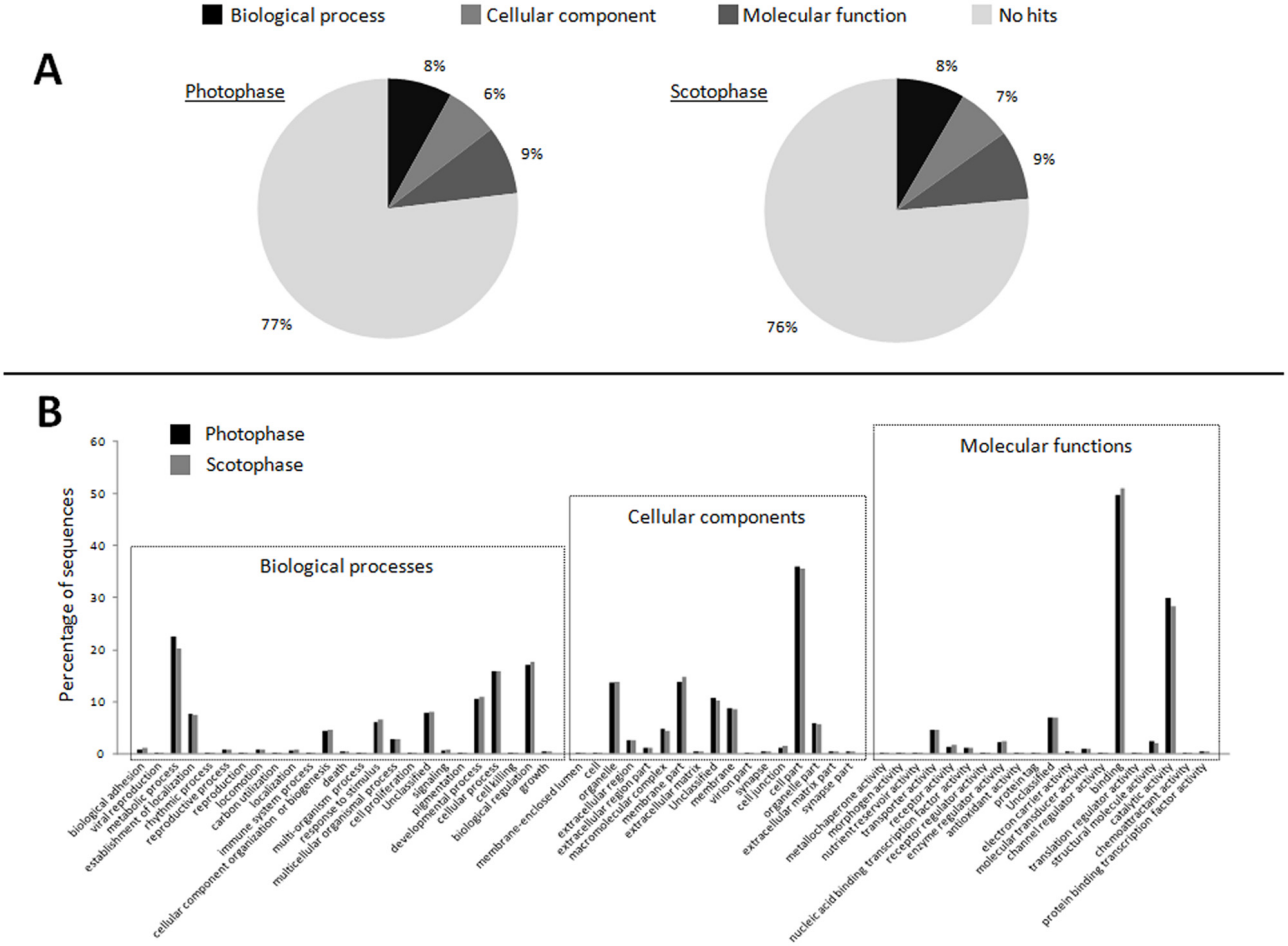
Gene ontology annotation of the two transcriptomes (NEP-L and NEP-D). Gene ontology (GO) annotation of the assembled transcriptomes. **A:** The percentage distribution of functional categories between the two transcriptomes together with the proportion on no hits. **B:** The percentage of sequences distributed among 62 different functional groups of both samples (black columns: photophase; grey columns: scotophase).

The sequencing of libraries produced a total of approximately 88 and 92 millions of paired-end reads for NEP-L and NEP-D respectively (Table 1; SRA accession SRP063649). The *de novo* assembly of NEP-Comb produced 108,599 contigs with a N50 (i.e. the size at which half of all assembled bases reside in contigs of this size or longer) of 1810. The *de novo* assembly of NEP-L produced 106,256 contigs with a N50 of 1,796, while for NEP-D the number of contigs was 114,235 with an N50 of 2,055. The species distribution of the annotated hits of transcripts of NEP-Comb against the NCBI non-redundant protein sequence database is presented in Fig 2. At about 73% of the first 30 species in order of number of annotated hits were insects, while the second species was the crustacean *Daphnia pulex*. The annotation of transcript sequences of NEP-L and NEP-D against the GO vocabulary produced 81,711 (77%) of no hits in NEP-L and 87,312 (76%) in NEP-D. The positive hits and following assignment of functional categories were distributed as follows: biological processes 8,522 (8%), cellular component 6,944 (6%), molecular function 9,079 (9%) for NEP-L; biological process 9,586 (8%), cellular component 7,583 (7%), molecular function 9,754 (9%) for NEP-D (Fig 3). The Fisher's exact test indicated that the 62 functional groups are equally represented among the two transcriptomes, so we reported the detailed percentage of GO annotation for both samples (Fig 3).

**Table 1:** Illumina sequencing. Descriptive statistics for the Illumina sequencing run and the assembly of the *de novo* transcriptomes.

RAW SEQUENCES	NEP-L	NEP-D	NEP-comb
Read type	Paired-end		-
Read length (bp)	101		-
Number of total reads	87'830'082	91'938'198	-
Total (bp)	8'870'838'282	9'285'757'998	-
**TRANSCRIPTOME ASSEMBLY**			
Total length of contigs	94'950'636	109'100'701	97'192'541
Total number of contigs	106'256	114'235	108'599
Max length	13'517	26'988	13'280
Min length	201	201	201
N90	311	322	310
N80	532	571	530
N70	864	956	875
N60	1'300	1'468	1'305
N50	1'796	2'055	1'810

The screening for putative clock genes produced 140 positive matches. Different sequences showed positive hits with genes related to the circadian system: *timeless*, *period*, *clock*, *cycle*, *bmal1*, *cryptochrome*, *double time*, *vrille*, *clockwork orange* and *jetlag*. Further details concerning the annotation are reported in the Table C in S1 Text, while the contigs are available in the Appendix B in S1 File.

**Fig 4:**
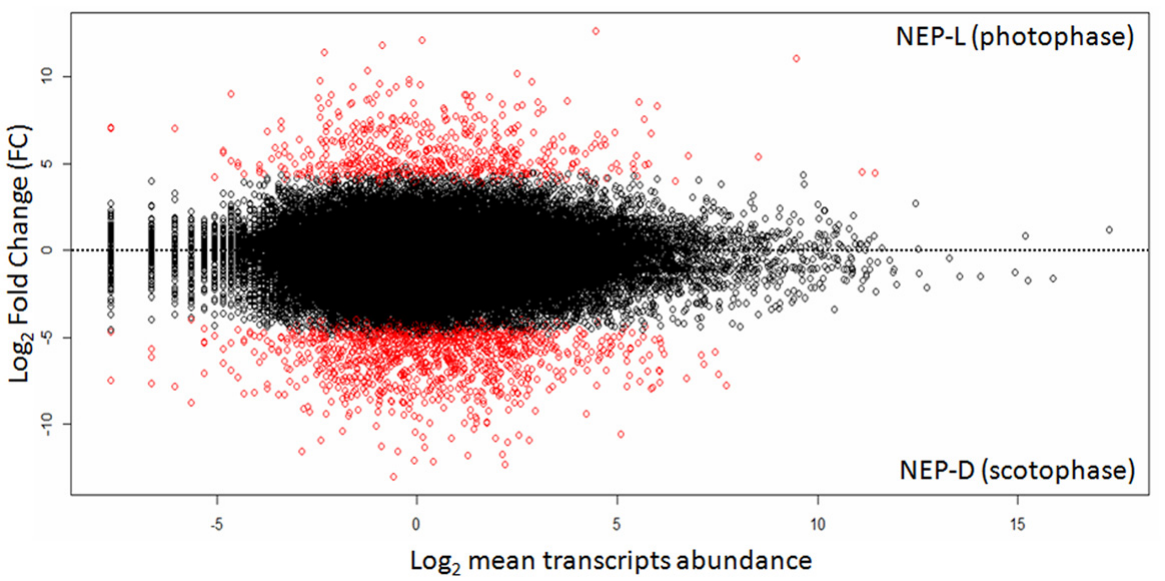
Fold change and abundance levels of transcripts. Different expression values of the two lobsters NEP-L and NEP-D. The black dots represent the equally expressed transcripts, while the red ones the differently expressed. The x-axis represents the Log_2_ of transcripts abundances. The y-axis represents the fold changes in abundances. In the upper part of the graph are plotted the transcripts of NEP-L and in the bottom part NEP-D.

The differential expression analysis indicated that 1182 transcripts are more expressed during scotophase, while 555 transcripts are more expressed during photophase. Fold changes are higher during scotophase than photophase (Table D in S1 Text). Regarding the genes related to the circadian system, only four of them have significant differences in levels of abundance (see Table C in S1 Text), and only one matched to an important *Drosophila* clock gene (*Dmel\timeless*). This transcript is more expressed during scotophase (fold change fold change L/D of 1.9·10^−2^). Finally, in Fig 4 is presented the global fold changes in all transcripts respect to the abundance levels.

### Cloning and quantitative real-time RT-PCR

The partial sequences of putative *Nephrops* clock genes obtained by cloning exhibited high levels of identity and similarity with the contigs obtained by the *de novo* assembly of the transcriptome (Table 2). We cloned a fragment of 4,754-bp for the putative *Nephrops period* gene (accession KP943777) that showed a 98.3% of similarity and 1.3% of gaps with the assembled contig on which we designed the primers. The cloned fragment for the putative *Nephrops timeless* gene (accession KP943778) had 2,137-bp and showed a 100% similarity. The fragments cloned for the *clock* (accession KP943779) and *bmal1* (accession KP943781) genes were shorter, 272-bp and 222-bp respectively, and had 100% similarity in both cases. We also cloned a 1036-bp fragment of the *Nephrops α-actin* gene (accession KP943780) that showed a 96.9% of similarity with the homologous gene of *H*. *americanus* with a 0.4% of gaps.

**Table 2:** Similarities between contigs and cloned fragments. Similarities observed during the alignment between the cloned sequences and the corresponding assembled contigs (*period*, *timeless*, *clock* and *bmal1*). For *α-act* the alignment was between the cloned sequence and the sequence retrieved from *H*. *americanus*.

Genes	Alignment	Length (bp)	Similarity (%)	Gaps (%)
*period*	assembling vs cloning	4754	98.3	1.3
*timeless*	assembling vs cloning	2137	100	0
*clock*	assembling vs cloning	272	100	0
*bmal1*	assembling vs cloning	222	100	0
*α-act*	*H*.*americanus vs N*.*norvegicus*	1036	96.9	0.4

The whole open reading frame of the contigs of the four putative clock genes were blasted on NCBI using the tool blastx to compare the translated protein products and results of representative best matches are reported in Table 3. The contigs were conceptually translated in peptides with the following amino acid (aa) lengths: PERIOD, 1654aa; TIMELESS, 842aa; CLOCK, 148aa; BMAL1, 74aa. *Nephrops* putative clock proteins showed higher levels of similarity with other crustaceans of the order Decapoda (e.g. the Signal crayfish, *Pacifastacus leniusculus* and the giant river prawn, *Macrobrachium rosenbergii*), Isopoda (e.g. the speckled sea louse, *Eurydice pulchra*) and with insects of different orders such as Diptera (e.g. the fruit fly, *Drosophila melanogaster*) and Orthoptera (e.g. the mangrove cricket, *Apteronemobius asahinai*).

**Table 3:** The most representative match of the blastx against NCBI database of the putative canonical clock genes of *Nephrops*.

Genes	Species	Phylum—Class -Order	Protein product	Identity	Gaps	Accession
*NnPeriod*	*Eurydice pulchra*	Arthropoda—Malacostraca—Isopoda	period	520/1101	110/1101	AGV28714
	*Blattella germanica*	Arthropoda—Insecta—Blattodea	circadian clock protein period	360/1099	167/1099	AAN02439
	*Apteronemobius asahinai*	Arthropoda—Insecta—Orthoptera	period isoform1	356/1100	157/1100	BAL72155
	*Laupala cerasina*	Arthropoda—Insecta—Orthoptera	period	317/1031	149/1031	ADO24376
	*Rhyparobia maderae*	Arthropoda—Insecta—Blattodea	period	201/538	46/538	AGA01525
*NnTimeless*	*Eurydice pulchra*	Arthropoda—Malacostraca—Isopoda	timeless	471/799	34/799	AGV28716
	*Thermobia domestica*	Arthropoda—Insecta—Thysanura	timeless	203/456	26/456	BAL27710
	*Drosophila melanogaster*	Arthropoda—Insecta—Diptera	timeless	196/465	13/465	AAC46920
	*Clunio marinus*	Arthropoda—Insecta—Diptera	timeless	192/456	20/456	AFS34623
	*Belgica antarctica*	Arthropoda—Insecta—Diptera	timeless	188/459	34/459	AGZ88039
*Nnclock*	*Pacifastacus leniusculus*	Arthropoda—Malacostraca—Decapoda	clock-like protein	34/55	15/55	AFV39704
	*Anopheles darlingi*	Arthropoda—Insecta—Diptera	clock-like protein	34/41	0/41	ETN62614
	*Macrobrachium rosenbergii*	Arthropoda—Malacostraca—Decapoda	clock	28/29	0/29	AAX44045
	*Thermobia domestica*	Arthropoda—Insecta—Thysanura	clock	25/29	0/29	AJ16353
	*Eurydice pulchra*	Arthropoda—Malacostraca—Isopoda	clock 1–7	25/29	0/29	AGV28721
*Nnbmal1*	*Pacifastacus leniusculus*	Arthropoda—Malacostraca—Decapoda	bmal1a	72/75	1/75	AFV39705
	*Eurydice pulchra*	Arthropoda—Malacostraca—Isopoda	brain and muscle arnt-like protein-1	59/75	1/75	AGV28715
	*Tribolium castaneum*	Arthropoda—Insecta—Coleoptera	cycle protein	46/73	6/73	EFA01256
	*Phyllotreta striolata*	Arthropoda—Insecta—Coleoptera	cycle protein, partial	46/61	6/61	CCA29756
	*Culex quinquefasciatus*	Arthropoda—Insecta—Diptera	circadian protein clock/arnt/bmal/pas	45/72	6/72	XP_001865023

We also blasted a contig (comp1618_c0_seq1; the contig is available in the Appendix B in S1 File) that was annotated against the GO database to *cryptochrome* (from *Homo sapiens*). The blastx of the contig against the NCBI database produced high level of identities with crustaceans of the Class Malacostraca, in particular with *cryptochrome* of *E*. *superba* (82%), and with *cryptochrome 2* of *E*. *pulchra* (79%) and *T*. *saltator* (79%) (see Table E in S1 Text).

The conceptual translation of canonical clock genes cDNAs indicated the presence of conserved domains (Table F in S1 Text). These were identified by SMART and blasted against NCBI database. *Nephrops* putative PERIOD has two PAS domains (from 229-296aa and from 373-442aa) and a PAC motif (from 450-493aa) that showed a high level of homology (expressed as identity) with conserved domains on the PERIOD protein of the isopod of *E*. *Pulchra* (PAS: 229-296aa, 85%; PAS: 373-442aa, 74%; PAC, 84%). *Nephrops* BMAL1 has the basic helix-loop-helix (bHLH, from 41-74aa) conserved domain that showed high level of homology with the bHLH domain on the protein BMAL1 of the decapod *P*. *leniusculus* (100%) and the isopod *E*. *Pulchra* (97%) (Table F in S1 Text). No conserved domains were identified in *Nephrops* TIMELESS and CLOCK.

**Fig 5:**
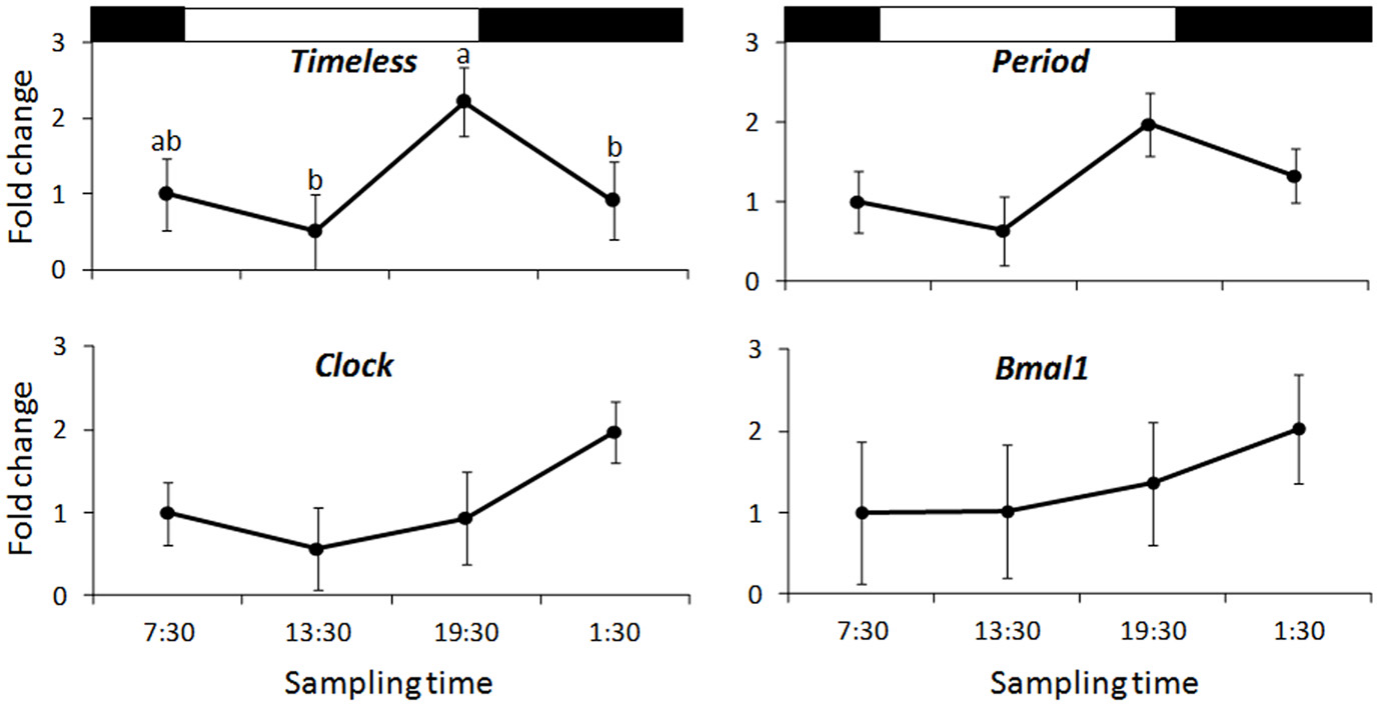
Canonical clock genes expression (RT-qPCR). Canonical clock genes expression in *Nephrops* eyestalk. Measurements (n = 3 each time point) were normalized to *α-act* and *18S* and expressed as fold change respect to a control time point (7:30). Vertical bars represent the confidence limits. Black and white bars represent darkness and light hours, respectively. *Timeless* shows a significant pattern of expression (ANOVA, P < 0.05). Letters indicate the output of the Tukey’s post hoc test (a>b).

The melting curves of the RT-qPCR indicated the presence of a single peak, suggesting no signs of contamination by DNA (as also supported by gel electrophoresis and absorbance ratio, see above). Among the canonical *Nephrops* clock genes, only *timeless* has a significant (F = 10.470, *P* < 0.01) pattern of expression with a peak just before the light-OFF (late day, Fig 5). The other transcripts did not show significant differences of expression among the different sampling time points: *period*, (F = 2.020, *p* = 0.19); *clock*, (F = 1.354; *p* = 0.32); *bmal1*, (F = 1.342, *p* = 0.33). Despite its lack of significant rhythmicity, *period* expression pattern appeared to be similar to *timeless*. The 2Δ*CT* values were used to assess differences among sampling times and are available in the Appendix C in the S1 File.

## Discussion

Here we assembled for the first time the eyestalk transcriptome of *Nephrops norvegicus* and annotated putative clock genes. We confirmed the fidelity of *de novo* assembly of canonical clock genes by cloning them. Conceptually translated protein products of partial fragments of *N*. *norvegicus period*, *timeless*, *clock and bmal1* showed high similarities with these genes in crustaceans and insects, with the presence of characteristics conserved domains (PAS and bHLH). Other putative homologous of clock genes include *cryptochrome 2*. Interestingly, results of the RT-qPCR experiment indicated that *timeless* oscillates with a diel rhythm and could be considered a suitable genetic marker of the molecular circadian clockwork controlling *Nephrops* locomotor activity rhythm. Together, our results are consistent with the notion that the eyestalk in decapod crustaceans houses a circadian oscillator involved in the regulation of behavioral and physiological rhythms.

The amount and quality of the reads produced for the present study are consistent with the average throughput produced by Illumina HiSeq 2000 platform. The assembly statistics are in line with those produced by other studies using a similar approach [45]. The similar distribution of the 62 functional groups during GO analysis between the light and dark phase of the LD cycle suggests that there is no difference in gene expression in terms of broad functional categories. The screening of the *de novo* assembled transcriptome identified 140 transcripts encoding for putative circadian proteins, demonstrating its power when applied to non-model species where scarce or absent previous genomic knowledge is available.

The similarities observed comparing the cloned and assembled cDNAs fragments of putative canonical clock genes of *Nephrops* validates a high fidelity of the assembly performed with trinity, as previously demonstrated in yeast, mouse and non-model organisms such as the whiteflies [33]. The blastx of the contigs encoding the putative clock genes of *Nephrops* against NCBI database revealed high identities with the translated proteins for the full length cDNAs sequences from *E*. *pulchra* (*period*: 47%, *timeless*: 59%, *bmal1*: 79%) and *M*. *rosenbergii (clock*: 100%*)*. The homology with other crustacean clock proteins strongly suggest that the partial fragments cloned in this study could be considered homologous of canonical clock genes and hence part of the transcriptional-translational feedback loop that constitutes the molecular circadian machinery in all metazoans studied so far [14]. This notion is reinforced by the presence of some characteristic conserved domains of clock proteins; *Nephrops* PERIOD has the PER-ARNT-SIM (PAS) domains, while *Nephrops* BMAL1 showed the basic helix-loop-helix (bHLH) domain. These domains are fundamental for the expression of protein PERIOD and TIMELESS and their activation by the heterodimeric bHLH-PAS transcription factors CLOCK and BMAL/CYCLE [46–48].

The transcripts retrieved in this study (Fig 4) give a global view of the differential expression of genes during the two opposite phases of burrow emergence. There are 1182 transcripts more expressed during scotophase and only 555 during photophase. *comp1372_c1_seq3 and comp1372_c1_seq1* are among the most expressed annotated transcripts during scotophase (fold change L/D of 2.0·10^−4^ and 4.8·10^−4^, respectively; see Table D in S1 Text); they matched with the gene *blent* of *D*. *melanogaster*. This gene is related to the structural constituent of cytoskeleton and a recent study demonstrates that is associated also with long-term memory (LTM) of courtship in *Drosophila* [49]. LTM seems to be independent by the core oscillator of the circadian clock even if *period* plays a key role in LTM formation [50]. The transcripts *comp259_c0_seq13* and *comp259_c0_seq19* are also more abundant during scotophase (fold change L/D of 2.3·10^−4^ and 5.6·10^−4^); they are annotated to the gene *myosin heavy chain* (*mhc*) of *D*. *melanogaster* that is associated with the striated muscle concentration and has been already observed to cycle under LD condition with a nocturnal phase [51]. The transcript *comp1307 c0 seq2* (fold change L/D of 2.9·10^−4^) matched to the gene *MSF3* of *D*. *melanogaster* which has a function in transmembrane transport and was recently considered as prime candidate transcript of newly circadian gene in pan-clock neurons of *Drosophila* [52, 53]. On the other hand, among the annotated transcripts that are more expressed during photophase, we found *comp2506_c0_seq2* (fold change L/D of 4.5·10^3^; see Table 3). This gene showed high similarity with the *D*. *melanogaster CG42327* that is implicated in protein dephosphorylation, an important molecular activity to maintain stable circadian oscillations [54]. Another transcript is *comp968_c1_seq4* (fold change L/D 2.7·10^3^) that matched to *D*. *melanogaster capulet* that has a function related to actin binding [55]. However, *capulet* seems to be also important for the transduction of photoperiodic signal [56].

Among the four genes studied with RT-qPCR only *timeless* had an oscillating pattern of expression. Our results are consistent with a recent study on *E*. *pulchra* where *period*, *timeless*, *bmal1*, *clock* and *cryptochrome 2* were studied using RT-qPCR [57]. Those authors reported that only *timeless* gave a robust and reliable circadian expression in whole head tissue, with a peak late in the subjective day. We did not expose lobsters to constant conditions, but previous studies have demonstrated that *Nephrops* burrow emergence is under the control of circadian system and can be entrained by blue light [58]. The peak of *timeless* transcripts is observed in lobsters sampled 30 min before light-OFF when animals also showed anticipation (increase of activity before any change in light intensity, see Fig 1), suggesting that the observed oscillation of *timeless* transcripts is endogenous. We also did not observe a significant diel pattern of expression for *clock*. Yang and colleagues [59] showed similar results for *M*. *rosembergii clock (Mar-clock)* under LD conditions; using semi-quantitative RT-PCR and *beta-actin* as internal control, the authors did not observed diel patterns of expression either in the central nervous system or peripheral tissues. However the expression tended to increase at night, as observed in our study. We have to mention that some of the RT-qPCR primers are designed on short gene sequences (e.g. *bmal1*) and this, of course, could alter the results. In fact, future studies should focus on the full length cloning of the clock genes characterized here.

Finally, a contig of 3,239 bp matched with the vertebrate-like *cryptochrome 2*. The blastx of this contig against the NCBI database produced high level of identities with other crustacean species such as *E*. *superba* (82%), *E*. *pulchra* (79%), *T*. *saltator* (79%) (see Table F in S1 Text). *Cryptochrome 2* was initially described for non-drosophilid insect species and proposed as a transcriptional repressor for the clock molecular machinery [60] Its expression has diel oscillations in *E*. *superba* both in laboratory [61] and in natural conditions [44]; recently it has been suggested as a major negative regulator also for the circadian clock of the crustacean *E*. *pulchra* [57]. Future studies could determine whether the same is true in *Nephrops*.

## Conclusions

We identified several putative clock genes in *Nephrops*. The finding that *timeless* is the only oscillating transcript for *Nephrops norvegicus* (at least in eyestalk) is consistent with the current knowledge on crustaceans’ circadian clocks, suggesting that the molecular clockwork of this group of arthropods may differ from that in *Drosophila*. This is also reinforced by the identification of a *Nephrops* homolog of the vertebrate-like *cryptochrome 2*. The results presented here, although preliminary, could become the basis for future research aimed at elucidating the crustacean molecular clockwork, with a particular emphasis on decapod crustaceans in the deep-water marine environment [62].

## Supporting Information

S1 Text **Appendix A. Calculation of the 2** ^**ΔΔ*CT***^ **; Appendix B. Calculation of the error propagation; Appendix C. Statistical analysis of RT-qPCR results; Table A. Primers used for cloning; Table B. Primers used for RT-qPCR; Table C. List of candidate clock genes in *Nephrops norvegicus***. Transcripts that can be considered as putative clock genes or genes related to the circadian system. **PPDE** (Posterior Probability of Differential Expression) represents the posterior probability of differential expression. **post FC (L/D)** represents the fold change of transcripts abundance between photophase/scotophase. Bold-highlighted transcripts result to be differentially expressed (PPDE > 0.95) across the two phases. (+) and (-) symbols indicate up- and down- regulation respectively during the light phase; **Table D. List of the representative transcripts that showed the highest levels of abundance during photophase and scotophase**. Trancripts were ordered by rank, together with the information related to their annotation (symbol of the hit, species, Go function and access number of the hit). PPDE (Posterior Probability of Differential Expression) represents the posterior probability of differential expression. Post FC (L/D) represents the fold change of transcripts abundance between photophase/scotophase; **Table E. Blastx of the contig annotated to *cryptochrome*; Table F. Conserved domains of canonical clock genes in *Nephrops norvegicus***.
(DOCX)

S1 File **Appendix A. Time series locomotor activity of lobsters**. Locomotor activity of lobsters expressed as cm travelled outside the burrow. The first line represents the ID of the lobster. Data are binned at 10 min and time series start at 00:00 (hh:mm); **Appendix B. Contigs from S1 Table C, D**. Contigs presented in Tables C and D in the S1 Text are available here; **Appendix C. 2ΔCT values**.
(ZIP)
