# Correction: Identification, Characterization, and Diel Pattern of Expression of Canonical Clock Genes in *Nephrops norvegicus* (Crustacea: Decapoda) Eyestalk

**DOI:** 10.1371/journal.pone.0200981

**Published:** 2018-07-16

**Authors:** The PLOS ONE Staff

There is an error in the XML that is causing the third author’s name, Audrey M. Mat, to be indexed incorrectly. The name should be indexed as Mat, Audrey M. The correct citation is: Sbragaglia V, Lamanna F, Mat AM, Rotllant G, Joly S, Ketmaier V, et al. (2015) Identification, Characterization, and Diel Pattern of Expression of Canonical Clock Genes in *Nephrops norvegicus* (Crustacea: Decapoda) Eyestalk. PLoS ONE 10(11): e0141893. https://doi.org/10.1371/journal.pone.0141893. The publisher apologizes for this error.

## References

[1] Sbragaglia V, Lamanna F, M. Mat A, Rotllant G, Joly S, Ketmaier V, et al (2015) Identification, Characterization, and Diel Pattern of Expression of Canonical Clock Genes in *Nephrops norvegicus* (Crustacea: Decapoda) Eyestalk. PLoS ONE 10(11): e0141893 10.1371/journal.pone.0141893 26524198 PMC4629887

