# Supplementary material for: Identification, Characterization, and Diel Pattern of Expression of Canonical Clock Genes in Nephrops norvegicus (Crustacea: Decapoda) Eyestalk
Source: PLoS One. 2015 Nov 2;10(11):e0141893. doi: 10.1371/journal.pone.0141893 (PMC4629887; doi:10.1371/journal.pone.0141893)
Supplement: S1 Text — Appendix A. Calculation of the 2 ΔΔCT ; Appendix B. Calculation of the error propagation; Appendix C. Statistical analysis of RT-qPCR results; Table A. Primers used for cloning; Table B. Primers used for RT-qPCR; Table C. List of candidate clock genes in Nephrops norvegicus. Transcripts that can be considered as putative clock genes or genes related to the circadian system. PPDE (Posterior Probability of Differential Expression) represents the posterior probability of differential expression. post FC (L/D) represents the fold change of transcripts abundance between photophase/scotophase. Bold-highlighted transcripts result to be differentially expressed (PPDE > 0.95) across the two phases. (+) and (-) symbols indicate up- and down- regulation respectively during the light phase; Table D. List of the representative transcripts that showed the highest levels of abundance during photophase and scotophase. Trancripts were ordered by rank, together with the information related to their annotation (symbol of the hit, species, Go function and access number of the hit). PPDE (Posterior Probability of Differential Expression) represents the posterior probability of differential expression. Post FC (L/D) represents the fold change of transcripts abundance between photophase/scotophase; Table E. Blastx of the contig annotated to cryptochrome; Table F. Conserved domains of canonical clock genes in Nephrops norvegicus. (DOCX) [file pone.0141893.s001.docx]

**Supporting Information**

**S1 Text. Appendix A. Calculation of the** $\boldsymbol{2}^{\boldsymbol{\Delta\Delta CT}}$

$\boldsymbol{\Delta\Delta}\boldsymbol{CT}\boldsymbol{=}\left( \boldsymbol{CT} \left( \boldsymbol{target}\boldsymbol{,}\boldsymbol{untreated} \right)\boldsymbol{-} \boldsymbol{CT} \left( \boldsymbol{ref}\boldsymbol{,}\boldsymbol{untreated} \right) \right)\boldsymbol{-}\boldsymbol{(}\boldsymbol{CT}\left( \boldsymbol{target}\boldsymbol{,}\boldsymbol{treated} \right)\boldsymbol{-}\boldsymbol{CT} \left( \boldsymbol{ref}\boldsymbol{,}\boldsymbol{treated} \right)\boldsymbol{)}$

Where:

$\boldsymbol{CT} \left( \boldsymbol{target}\boldsymbol{,}\boldsymbol{untreated} \right)\boldsymbol{=}$ Ct value of the gene of interest in control sample (for convention time 7:30 in our study)

$\boldsymbol{CT} \left( \boldsymbol{ref}\boldsymbol{,}\boldsymbol{untreated} \right)\boldsymbol{=}$ Ct value of the geometric mean of the two control genes used in the control sample

$\boldsymbol{CT}\left( \boldsymbol{target}\boldsymbol{,}\boldsymbol{treated} \right)\boldsymbol{=}$ Ct value of the gene of interest at the different time points

$\boldsymbol{CT} \left( \boldsymbol{ref}\boldsymbol{,}\boldsymbol{treated} \right)\boldsymbol{=}$ Ct value of the geometric mean of the two control genes at the different time points

We also calculated the $\boldsymbol{E}^{\boldsymbol{\Delta\Delta CT}}$

Where E represents the efficiency calculated as follow:

$$\boldsymbol{Efficiency}\boldsymbol{=}\boldsymbol{2}^{\boldsymbol{-1}\boldsymbol{/}\boldsymbol{slope}}$$

**S1 Text. Appendix B. Calculation of the error propagation**

The errors bars represented in Figure 5 (confidence limits) are calculated as follow for each single time point:

$$\boldsymbol{Error}\boldsymbol{=}\sqrt{\boldsymbol{(}\boldsymbol{Error}{\left( \boldsymbol{control} \right)\boldsymbol{)}}^{\boldsymbol{2}}\boldsymbol{+ (}\boldsymbol{Error}{\left( \boldsymbol{treated} \right)\boldsymbol{)}}^{\boldsymbol{2}}}$$

Remember that the control sample is at 7:30 time point for convention. For simplicity we report just the equation for the calculation of the error propagation at one representative time point (T)

$$\boldsymbol{Error}\left( \boldsymbol{T} \right)\boldsymbol{=}\sqrt{{\boldsymbol{(}\boldsymbol{Error}\left( \boldsymbol{ref} \right)\boldsymbol{)}}^{\boldsymbol{2}}\boldsymbol{+ (}{\boldsymbol{Error}\left( \boldsymbol{target} \right)\boldsymbol{)}}^{\boldsymbol{2}}}$$

$$\boldsymbol{Error} \left( \boldsymbol{ref} \right)\boldsymbol{=}\left( \boldsymbol{2}\boldsymbol{*}\boldsymbol{geom}\boldsymbol{.}\boldsymbol{mean}\left( \boldsymbol{ref}\boldsymbol{1}\boldsymbol{,}\boldsymbol{2} \right) \right)\boldsymbol{*} \sqrt{\left( \boldsymbol{arit}\boldsymbol{.}\boldsymbol{mean}\boldsymbol{(}\boldsymbol{ref}\boldsymbol{1}\boldsymbol{)}\boldsymbol{*}\boldsymbol{SE} \right)^{\boldsymbol{2}}\boldsymbol{+}\left( \boldsymbol{arit}\boldsymbol{.}\boldsymbol{mean}\left( \boldsymbol{ref}\boldsymbol{2} \right)\boldsymbol{*}\boldsymbol{SE} \right)^{\boldsymbol{2}}}$$

$$\boldsymbol{Error} \left( \boldsymbol{target} \right)\boldsymbol{=}\boldsymbol{standard} \boldsymbol{error} \boldsymbol{of} \boldsymbol{the} \boldsymbol{aritm}\boldsymbol{.}\boldsymbol{mean}\boldsymbol{=}\frac{\boldsymbol{Standard} \boldsymbol{deviation}}{\sqrt{\boldsymbol{n}}}$$

**S1 Text. Appendix C. Statistical analysis of RT-qPCR results**

A one way ANOVA was used to assess significant differences among RNA expression at different time point.

We used the $2\Delta CT$values as input for the statistical test. These were calculated as follow:

$$\boldsymbol{2}\boldsymbol{\Delta}\boldsymbol{CT}\boldsymbol{=}\boldsymbol{E}^{\boldsymbol{(}\left( \boldsymbol{-}\boldsymbol{1} \right)\boldsymbol{*}\boldsymbol{\Delta}\boldsymbol{CT}\boldsymbol{)}}$$

$$\boldsymbol{\Delta}\boldsymbol{CT}\boldsymbol{=}\boldsymbol{Mean} \boldsymbol{CT} \left( \boldsymbol{target}_{\boldsymbol{i}} \right)\boldsymbol{-} \boldsymbol{geom}\boldsymbol{.}\boldsymbol{mean} \boldsymbol{CT}\boldsymbol{(}\boldsymbol{ref}_{\boldsymbol{i}}\boldsymbol{1}\boldsymbol{,}\boldsymbol{2}\boldsymbol{)}$$

Where E = Efficiency as reported in Section 1

${\boldsymbol{CT} \boldsymbol{ref}}_{\boldsymbol{i}}\boldsymbol{1}\boldsymbol{,}\boldsymbol{2}\boldsymbol{=}$The CTs values of the *i* sample of cDNA for reference gene 1 and 2.

**S1 Text. Table A. Primers used for cloning**

| **Genes** | **Primer** | **Position (bp)** | **Primer sequence (5' -> 3')** | **Ann. Temp. (°C)** |
| --- | --- | --- | --- | --- |
| *period* | *per-F1* | 12-37 | CCCAGAGTTAGTGGAGTAAAGGTGTG | 56.7 |
|  | *per-R1* | 1262-1241 | TGTGGCAGATGACCCAGGTAGG |  |
|  | *per-F2* | 315-337 | TGGCGACAATGCTGATTTTAGTG | 57.2 |
|  | *per-R2* | 2103-2078 | TGAGAGAGTCTGTGAGTGTGATAGCG |  |
|  | *per-F3* | 2020-2039 | AGCACCCATCCAGCCTTTTC | 57.8 |
|  | *per-R3* | 3260-3238 | CGGTTCATCTCAGAATCCTTTGG |  |
|  | *per-F4* | 3058-3077 | CGTTCCACCACTAACCTGCG | 56.1 |
|  | *per-R4* | 4095-4073 | CCTTGAGCCACCTATTGCCATAC |  |
|  | *per-F5* | 3658-3678 | AGATTACGACAGCCTGCCTGG | 54.1 |
|  | *per-R5* | 4850-4831 | TCCGTTCTTTTTTTTCGCCC |  |
| *timeless* | *tim-F2* | 752-773 | TCGGACAGTTGGTAGAGGTGCG | 56.5 |
|  | *tim-R2* | 2304-2283 | TGTTTGAGGATTCGTCGTCGTG |  |
|  | *tim-F3* | 168-192 | GCACCATCAGAAGCCTCATAAAATG | 56.4 |
|  | *tim-R3* | 801-778 | CAAGCGAATCAGCAACACAAATAG |  |
| *clock* | *clk-F3* | 122-149 | AGTTTAGTGATAACCAGGGAGTAAGAGC | 54.5 |
|  | *clk-R3* | 393-372 | CGGACAGTTCGTTGATGAGGAG |  |
| *bmal1* | *bmal1-F1* | 1-24 | TCCTTCTCCTCTGATGGCTCTAAG | 52.1 |
|  | *bmal1-R1* | 222-200 | TGTCAGTTTGTCAAGCTTCCGAG |  |
| *α-act* | *a-act-F1* | 300-322 | CACTCCTTCTACAACGAACTGCG | 58.1 |
|  | *a-act-R1* | 1160-1141 | GCACTTGCGGTGGACAATGC |  |
|  | *a-act-F3* | 127-146 | TATTCCCCTCCATCGTCGGC | 57.7 |
|  | *a-act-R3* | 398-378 | GGTCATCTTCTCACGGTTGGC |  |

**S1 Text. Table B. Primers used for RT-qPCR**

| **Genes** | **Primer name** | **Position on cDNA fragment** | **Primer sequence (5' -> 3')** | **Amplicon size (bp)** | **Efficiency (E)** | **R²** |
| --- | --- | --- | --- | --- | --- | --- |
| *NnPeriod* | per-F1 | 364-388 | TGGAAGAAGTTGAAGGAGAAGACCG | 151 | 2.2 | 0.969 |
|  | per-R1 | 515-493 | CAATACTGCTGGCTGTTTCGCTG |  |  |  |
| *NnTimeless* | tim-F1 | 256-280 | GCCCTATCAGATTGACCTGGACAAG | 154 | 1.9 | 0.969 |
|  | tim-R1 | 409-385 | CATCACCACTCCCTCATACACCAAG |  |  |  |
| *Nnclock* | clk-F1 | 33-57 | TGGTGGTGGTGTGAAGTGGATTTAC | 132 | 2.5 | 0.970 |
|  | clk-R1 | 164-142 | CAGATTTGCCAGGTGATGTTTCG |  |  |  |
| *Nnbmal1* | bmal1-F1 | 1-21 | TCCTTCTCCTCTGATGGCTCT | 108 | 2.2 | 0.968 |
|  | bmal1-R1 | 108-89 | TTTATTCCAATCCCCAGCAG |  |  |  |
| *Nnα-act* | aAct-F2 | 414-438 | GGTTATTGTCTCCCACACGCTATCC | 136 | 2.0 | 0.979 |
|  | aAct-R2 | 549-527 | TGATGTCACGAACGATTTCTCGC |  |  |  |
| *Nn18S* | 18S-F1 | 132-153 | AGGGCGTTGGTCGTCGTTATTC | 134 | 2.0 | 0.968 |
|  | 18S-R1 | 265-246 | CTTGTATTGGCGGGGGTTGC |  |  |  |

**S1 Text. Table C. List of candidate clock genes in *Nephrops norvegicus.*** Transcripts that can be considered as putative clock genes or genes related to the circadian system. **PPDE** (Posterior Probability of Differential Expression) represents the posterior probability of differential expression. **post FC (L/D)** represents the fold change of transcripts abundance between photophase/scotophase. Bold-highlighted transcripts result to be differentially expressed (PPDE > 0.95) across the two phases. (+) and (-) symbols indicate up- and down- regulation respectively during the light phase.

| **Transcript** | **Length (bp)** | **Match** | **Start** | **End** | **PPDE** | **Post FC (L/D)** | **Species** | **Access number** |
| --- | --- | --- | --- | --- | --- | --- | --- | --- |
| comp65743_c0_seq1 | 304 | 48 related 2 | 3 | 299 | 0.125 | 0.301 | *Drosophila melanogaster* | FB\|FBgn0038402 |
| comp8903_c0_seq1 | 165 | 5'-AMP-activated protein kinase catalytic subunit alpha-2 | 135 | 1646 | 0.293 | 0.177 | *Pongo abelii* | UNIPROTKB\|Q5RD00 |
| comp6931_c0_seq1 | 3892 | Alpha-N-acetylglucosaminidase | 3549 | 1936 | 0.012 | 1.474 | *Homo sapiens* | UNIPROTKB\|P54802 |
| comp6931_c0_seq2 | 3710 | Alpha-N-acetylglucosaminidase | 3255 | 1936 | 0.13 | 3.121 | *Homo sapiens* | UNIPROTKB\|P54802 |
| comp48442_c0_seq1 | 219 | AT5G63860 | 2 | 193 | 0.083 | 0.462 | *rabidopsis thaliana* | TAIR\|locus:2163986 |
| comp1846_c0_seq1 | 6297 | ATP-binding cassette, sub-family B (MDR/TAP), member 1A | 352 | 3969 | 0.378 | 6.492 | *Rattus norvegicus* | RGD\|619951 |
| comp1846_c0_seq10 | 2755 | ATP-binding cassette, sub-family B (MDR/TAP), member 1A | 54 | 1505 | 0.055 | 2.461 | *Rattus norvegicus* | RGD\|619951 |
| comp1846_c0_seq11 | 2300 | ATP-binding cassette, sub-family B (MDR/TAP), member 1A | 733 | 1050 | 0.144 | 0.265 | *Rattus norvegicus* | RGD\|619951 |
| comp1846_c0_seq12 | 1899 | ATP-binding cassette, sub-family B (MDR/TAP), member 1A | 137 | 649 | 0.472 | 5.6 | *Rattus norvegicus* | RGD\|619951 |
| comp1846_c0_seq2 | 5387 | ATP-binding cassette, sub-family B (MDR/TAP), member 1A | 352 | 4137 | 0.927 | 0.062 | *Rattus norvegicus* | RGD\|619951 |
| comp1846_c0_seq5 | 3804 | ATP-binding cassette, sub-family B (MDR/TAP), member 1A | 517 | 1476 | 0.097 | 2.804 | *Rattus norvegicus* | RGD\|619951 |
| **comp1846_c0_seq6 (+)** | 3665 | ATP-binding cassette, sub-family B (MDR/TAP), member 1A | 54 | 1337 | 0.985 | 28.033 | *Rattus norvegicus* | RGD\|619951 |
| comp1846_c0_seq8 | 2894 | ATP-binding cassette, sub-family B (MDR/TAP), member 1A | 517 | 1644 | 0.048 | 0.848 | *Rattus norvegicus* | RGD\|619951 |
| comp65830_c0_seq1 | 855 | beta-transducin repeat containing | 2 | 853 | 0.369 | 0.166 | *Rattus norvegicus* | RGD\|1359721 |
| comp1689_c0_seq1 | 5472 | cAMP-dependent protein kinase 1 | 670 | 1614 | 0.02 | 0.686 | *Drosophila melanogaster* | FB\|FBgn0000273 |
| comp1689_c0_seq2 | 5177 | cAMP-dependent protein kinase 1 | 267 | 1319 | 0.006 | 0.926 | *Drosophila melanogaster* | FB\|FBgn0000273 |
| comp33327_c0_seq1 | 1296 | cAMP-dependent protein kinase R2 | 1156 | 44 | 0.083 | 0.458 | *Drosophila melanogaster* | FB\|FBgn0022382 |
| comp78077_c0_seq1 | 983 | Caveolin-1 | 947 | 459 | 0.197 | 3.621 | *Ornithorhynchus anatinus* | UNIPROTKB\|Q07E02 |
| comp30303_c0_seq1 | 535 | CG2650 | 510 | 91 | 0.06 | 0.839 | *Drosophila melanogaster* | FB\|FBgn0000092 |
| comp1595_c0_seq13 | 3033 | circadian trip | 1616 | 426 | 0.826 | 0.097 | *Drosophila melanogaster* | FB\|FBgn0260794 |
| comp1595_c0_seq14 | 3021 | circadian trip | 1616 | 426 | 0.351 | 0.184 | *Drosophila melanogaster* | FB\|FBgn0260794 |
| comp23855_c0_seq2 | 445 | Clock | 122 | 33 | 0.096 | 0.574 | *Drosophila melanogaster* | FB\|FBgn0023076 |
| comp10496_c0_seq1 | 3957 | clockwork orange | 2893 | 2486 | 0.035 | 1.343 | *Drosophila melanogaster* | FB\|FBgn0259938 |
| comp10496_c0_seq2 | 3936 | clockwork orange | 2872 | 2465 | 0.035 | 0.906 | *Drosophila melanogaster* | FB\|FBgn0259938 |
| comp10496_c0_seq3 | 3242 | clockwork orange | 3049 | 2486 | 0.025 | 0.808 | *Drosophila melanogaster* | FB\|FBgn0259938 |
| comp10496_c0_seq4 | 3221 | clockwork orange | 3028 | 2465 | 0.029 | 0.789 | *Drosophila melanogaster* | FB\|FBgn0259938 |
| comp1618_c0_seq1 | 3239 | Cryptochrome-1 | 197 | 1666 | 0.008 | 0.592 | *Homo sapiens* | UNIPROTKB\|Q16526 |
| comp25352_c0_seq1 | 1976 | curled | 231 | 1316 | 0.058 | 1.981 | *Drosophila melanogaster* | FB\|FBgn0261808 |
| comp25352_c0_seq2 | 1786 | curled | 83 | 1126 | 0.053 | 1.197 | *Drosophila melanogaster* | FB\|FBgn0261808 |
| comp25352_c0_seq3 | 1429 | curled | 302 | 769 | 0.065 | 0.817 | *Drosophila melanogaster* | FB\|FBgn0261808 |
| comp25352_c0_seq4 | 980 | curled | 231 | 854 | 0.842 | 0.066 | *Drosophila melanogaster* | FB\|FBgn0261808 |
| comp54873_c0_seq1 | 222 | cycle | 55 | 222 | 0.083 | 0.462 | *Drosophila melanogaster* | FB\|FBgn0023094 |
| comp1099_c0_seq1 | 7845 | discs large 1 | 1033 | 3504 | 0.006 | 0.536 | *Drosophila melanogaster* | FB\|FBgn0001624 |
| comp1099_c0_seq2 | 7827 | discs large 1 | 1033 | 3486 | 0.008 | 1.25 | *Drosophila melanogaster* | FB\|FBgn0001624 |
| comp21486_c0_seq1 | 706 | discs large 1 | 379 | 134 | 0.512 | 0.154 | *Drosophila melanogaster* | FB\|FBgn0001624 |
| comp16468_c0_seq1 | 6864 | Dopa decarboxylase | 6738 | 5344 | 0.019 | 0.753 | *Drosophila melanogaster* | FB\|FBgn0000422 |
| comp16468_c0_seq3 | 3218 | Dopa decarboxylase | 3092 | 1698 | 0.046 | 0.703 | *Drosophila melanogaster* | FB\|FBgn0000422 |
| comp49424_c0_seq1 | 527 | Dopamine transporter | 526 | 14 | 0.104 | 0.446 | *Drosophila melanogaster* | FB\|FBgn0034136 |
| comp69404_c0_seq1 | 229 | Dopamine transporter | 21 | 155 | 0.083 | 0.462 | *Drosophila melanogaster* | FB\|FBgn0034136 |
| comp73842_c0_seq1 | 482 | Dopamine transporter | 480 | 4 | 0.178 | 0.223 | *Drosophila melanogaster* | FB\|FBgn0034136 |
| comp27457_c0_seq1 | 3891 | Dttg protein | 439 | 837 | 0.038 | 0.76 | *Drosophila* sp. | UNIPROTKB\|P91608 |
| comp27457_c0_seq2 | 3828 | Dttg protein | 439 | 915 | 0.038 | 0.822 | *Drosophila* sp. | UNIPROTKB\|P91608 |
| comp11431_c0_seq1 | 4284 | dunce | 267 | 2375 | 0.094 | 0.574 | *Drosophila melanogaster* | FB\|FBgn0000479 |
| comp11431_c0_seq2 | 4263 | dunce | 267 | 2354 | 0.066 | 0.528 | *Drosophila melanogaster* | FB\|FBgn0000479 |
| comp11431_c0_seq3 | 4260 | dunce | 267 | 2351 | 0.189 | 0.293 | *Drosophila melanogaster* | FB\|FBgn0000479 |
| comp11431_c0_seq4 | 4239 | dunce | 267 | 2330 | 0.116 | 0.285 | *Drosophila melanogaster* | FB\|FBgn0000479 |
| comp11431_c0_seq5 | 3575 | dunce | 116 | 1666 | 0.05 | 1.279 | *Drosophila melanogaster* | FB\|FBgn0000479 |
| comp11431_c0_seq6 | 3554 | dunce | 116 | 1645 | 0.027 | 1.438 | *Drosophila melanogaster* | FB\|FBgn0000479 |
| comp12887_c0_seq1 | 1705 | dusky | 1464 | 676 | 0.02 | 1.611 | *Drosophila melanogaster* | FB\|FBgn0004511 |
| comp89314_c0_seq1 | 408 | dusky | 102 | 365 | 0.12 | 0.405 | *Drosophila melanogaster* | FB\|FBgn0004511 |
| **comp9690_c0_seq16 (-)** | 2331 | ebony | 596 | 2200 | 0.957 | 0.042 | *Drosophila melanogaster* | FB\|FBgn0000527 |
| comp9690_c0_seq22 | 1692 | ebony | 432 | 1061 | 0.151 | 0.256 | *Drosophila melanogaster* | FB\|FBgn0000527 |
| comp9690_c0_seq27 | 802 | ebony | 76 | 171 | 0.927 | 0.060 | *Drosophila melanogaster* | FB\|FBgn0000527 |
| comp9690_c0_seq6 | 3245 | ebony | 1738 | 3114 | 0.033 | 1.079 | *Drosophila melanogaster* | FB\|FBgn0000527 |
| comp9690_c0_seq8 | 3022 | ebony | 432 | 2891 | 0.036 | 0.449 | *Drosophila melanogaster* | FB\|FBgn0000527 |
| comp9690_c0_seq9 | 2739 | ebony | 1277 | 2608 | 0.155 | 0.345 | *Drosophila melanogaster* | FB\|FBgn0000527 |
| comp43073_c0_seq1 | 731 | Ecdysone receptor | 452 | 75 | 0.643 | 0.104 | *Drosophila melanogaster* | FB\|FBgn0000546 |
| comp45891_c0_seq1 | 264 | Ecdysone receptor | 263 | 159 | 0.083 | 0.462 | *Drosophila melanogaster* | FB\|FBgn0000546 |
| comp50197_c0_seq1 | 448 | Ecdysone receptor | 1 | 420 | 0.102 | 0.525 | *Drosophila melanogaster* | FB\|FBgn0000546 |
| comp59204_c0_seq1 | 513 | F-box and WD repeat domain containing 11 | 7 | 513 | 0.077 | 0.934 | *Rattus norvegicus* | RGD\|1309121 |
| comp8570_c0_seq1 | 3139 | glass | 1887 | 925 | 0.057 | 0.289 | *Drosophila melanogaster* | FB\|FBgn0004618 |
| comp11966_c0_seq1 | 820 | Heat shock protein 83 | 2 | 820 | 0.029 | 0.774 | *Drosophila melanogaster* | FB\|FBgn0001233 |
| comp19340_c0_seq1 | 2213 | jetlag | 2174 | 1398 | 0.026 | 1.072 | *Drosophila melanogaster* | FB\|FBgn0031652 |
| comp83131_c0_seq1 | 291 | KaiRIA | 1 | 291 | 0.089 | 2.289 | *Drosophila melanogaster* | FB\|FBgn0028422 |
| comp4180_c0_seq3 | 4236 | Large proline-rich protein BAG6 | 1313 | 87 | 0.059 | 0.51 | *Ornithorhynchus anatinus* | UNIPROTKB\|A7X5R6 |
| comp10165_c0_seq1 | 1259 | lark | 1258 | 575 | 0.059 | 1.76 | *Drosophila melanogaster* | FB\|FBgn0011640 |
| comp10165_c0_seq2 | 1227 | lark | 1226 | 543 | 0.062 | 2.021 | *Drosophila melanogaster* | FB\|FBgn0011640 |
| comp10165_c0_seq3 | 1185 | lark | 1123 | 575 | 0.143 | 0.377 | *Drosophila melanogaster* | FB\|FBgn0011640 |
| comp10165_c0_seq4 | 1153 | lark | 1091 | 543 | 0.251 | 0.251 | *Drosophila melanogaster* | FB\|FBgn0011640 |
| comp10165_c0_seq5 | 1095 | lark | 1094 | 498 | 0.154 | 3.774 | *Drosophila melanogaster* | FB\|FBgn0011640 |
| comp10165_c0_seq6 | 1063 | lark | 1062 | 466 | 0.09 | 1.742 | *Drosophila melanogaster* | FB\|FBgn0011640 |
| comp10165_c0_seq7 | 1021 | lark | 959 | 498 | 0.822 | 12.99 | *Drosophila melanogaster* | FB\|FBgn0011640 |
| comp10165_c0_seq8 | 989 | lark | 927 | 466 | 0.074 | 0.448 | *Drosophila melanogaster* | FB\|FBgn0011640 |
| comp10165_c0_seq9 | 335 | lark | 334 | 212 | 0.089 | 0.378 | *Drosophila melanogaster* | FB\|FBgn0011640 |
| comp1808_c0_seq1 | 1440 | lark | 101 | 688 | 0.008 | 0.853 | *Drosophila melanogaster* | FB\|FBgn0011640 |
| comp1808_c0_seq2 | 902 | lark | 101 | 301 | 0.024 | 1.298 | *Drosophila melanogaster* | FB\|FBgn0011640 |
| comp29701_c0_seq1 | 1374 | lark | 1112 | 639 | 0.036 | 0.988 | *Drosophila melanogaster* | FB\|FBgn0011640 |
| comp6509_c0_seq1 | 1127 | lark | 471 | 115 | 0.059 | 1.997 | *Drosophila melanogaster* | FB\|FBgn0011640 |
| comp6509_c0_seq2 | 959 | lark | 303 | 1 | 0.032 | 0.792 | *Drosophila melanogaster* | FB\|FBgn0011640 |
| comp6509_c0_seq3 | 747 | lark | 471 | 115 | 0.332 | 0.157 | *Drosophila melanogaster* | FB\|FBgn0011640 |
| comp6509_c0_seq4 | 579 | lark | 303 | 1 | 0.138 | 2.73 | *Drosophila melanogaster* | FB\|FBgn0011640 |
| comp3068_c0_seq1 | 3907 | minibrain | 1730 | 2935 | 0.323 | 0.18 | *Drosophila melanogaster* | FB\|FBgn0259168 |
| comp3068_c0_seq2 | 3864 | minibrain | 1730 | 2935 | 0.051 | 0.895 | *Drosophila melanogaster* | FB\|FBgn0259168 |
| comp5354_c0_seq1 | 3603 | Myocyte enhancer factor 2 | 71 | 1216 | 0.024 | 0.794 | *Drosophila melanogaster* | FB\|FBgn0011656 |
| comp5354_c0_seq2 | 3591 | Myocyte enhancer factor 2 | 71 | 1237 | 0.011 | 0.866 | *Drosophila melanogaster* | FB\|FBgn0011656 |
| comp34215_c0_seq1 | 967 | narrow abdomen | 2 | 484 | 0.073 | 0.591 | *Drosophila melanogaster* | FB\|FBgn0002917 |
| comp66820_c0_seq1 | 451 | narrow abdomen | 451 | 2 | 0.307 | 0.147 | *Drosophila melanogaster* | FB\|FBgn0002917 |
| comp85336_c0_seq1 | 285 | narrow abdomen | 37 | 285 | 0.125 | 0.301 | *Drosophila melanogaster* | FB\|FBgn0002917 |
| comp96661_c0_seq1 | 223 | narrow abdomen | 221 | 3 | 0.083 | 0.462 | *Drosophila melanogaster* | FB\|FBgn0002917 |
| comp97497_c0_seq1 | 329 | narrow abdomen | 2 | 328 | 0.083 | 0.462 | *Drosophila melanogaster* | FB\|FBgn0002917 |
| comp8495_c0_seq1 | 8857 | Neurofibromin 1 | 65 | 7399 | 0.079 | 0.587 | *Drosophila melanogaster* | FB\|FBgn0015269 |
| comp8495_c0_seq2 | 8845 | Neurofibromin 1 | 65 | 7387 | 0.046 | 0.721 | *Drosophila melanogaster* | FB\|FBgn0015269 |
| comp8495_c0_seq3 | 8686 | Neurofibromin 1 | 65 | 7399 | 0.046 | 0.48 | *Drosophila melanogaster* | FB\|FBgn0015269 |
| comp8495_c0_seq4 | 8674 | Neurofibromin 1 | 65 | 7387 | 0.02 | 0.616 | *Drosophila melanogaster* | FB\|FBgn0015269 |
| **Comp841_c0_seq1 (-)** | 4029 | no receptor potential A | 474 | 3737 | 0.973 | 0.036 | *Drosophila melanogaster* | FB\|FBgn0262738 |
| comp8804_c0_seq1 | 2651 | numb | 281 | 1441 | 0.076 | 1.612 | *Drosophila melanogaster* | FB\|FBgn0002973 |
| comp8804_c0_seq2 | 2614 | numb | 244 | 1404 | 0.058 | 1.576 | *Drosophila melanogaster* | FB\|FBgn0002973 |
| comp8804_c0_seq3 | 2585 | numb | 281 | 1375 | 0.052 | 0.555 | *Drosophila melanogaster* | FB\|FBgn0002973 |
| comp8804_c0_seq4 | 2573 | numb | 203 | 1363 | 0.103 | 1.857 | *Drosophila melanogaster* | FB\|FBgn0002973 |
| comp8804_c0_seq5 | 2548 | numb | 244 | 1338 | 0.031 | 0.658 | *Drosophila melanogaster* | FB\|FBgn0002973 |
| comp8804_c0_seq6 | 2507 | numb | 203 | 1297 | 0.072 | 0.674 | *Drosophila melanogaster* | FB\|FBgn0002973 |
| comp2789_c0_seq1 | 4961 | period | 4220 | 2901 | 0.032 | 0.384 | *Drosophila melanogaster* | FB\|FBgn0003068 |
| comp24010_c0_seq1 | 1436 | Pigment-dispersing factor receptor | 81 | 1259 | 0.034 | 0.838 | *Drosophila melanogaster* | FB\|FBgn0260753 |
| comp33631_c0_seq1 | 4066 | Pigment-dispersing factor receptor | 722 | 2005 | 0.038 | 0.554 | *Drosophila melanogaster* | FB\|FBgn0260753 |
| comp2319_c0_seq1 | 621 | Prokineticin-2 | 425 | 177 | 0.661 | 7.021 | *Bos taurus* | UNIPROTKB\|Q863H5 |
| comp2319_c0_seq2 | 578 | Prokineticin-2 | 382 | 134 | 0.085 | 3.765 | *Bos taurus* | UNIPROTKB\|Q863H5 |
| comp17940_c0_seq1 | 1678 | Protein lin-52 homolog | 315 | 551 | 0.745 | 0.107 | *Oncorhynchus mykiss* | UNIPROTKB\|Q6X4M3 |
| comp17940_c0_seq2 | 1573 | Protein lin-52 homolog | 105 | 446 | 0.037 | 1.177 | *Oncorhynchus mykiss* | UNIPROTKB\|Q6X4M3 |
| comp17014_c0_seq1 | 700 | Protein quiver | 274 | 576 | 0.241 | 0.271 | *Drosophila mojavensis* | UNIPROTKB\|B4KR21 |
| comp17014_c0_seq2 | 492 | Protein quiver | 277 | 492 | 0.109 | 0.317 | *Drosophila mojavensis* | UNIPROTKB\|B4KR21 |
| comp43724_c0_seq1 | 2037 | Protein timeless homolog | 1732 | 161 | 0.043 | 1.556 | *Homo sapiens* | UNIPROTKB\|Q9UNS1 |
| comp10628_c0_seq1 | 2883 | quasimodo | 459 | 1292 | 0.03 | 2.77 | *Drosophila melanogaster* | FB\|FBgn0028622 |
| comp62503_c0_seq1 | 366 | Rhythmically expressed gene 5 | 226 | 336 | 0.176 | 3.444 | *Drosophila melanogaster* | FB\|FBgn0015801 |
| comp54599_c0_seq1 | 573 | RNA-binding protein 4B | 572 | 279 | 0.086 | 0.58 | *Sus scrofa* | UNIPROTKB\|F1RUT7 |
| comp573_c0_seq1 | 3368 | RNA-binding protein 4B | 129 | 371 | 0.015 | 1.059 | *Sus scrofa* | UNIPROTKB\|F1RUT7 |
| comp573_c0_seq2 | 3044 | RNA-binding protein 4B | 129 | 371 | 0.011 | 1.192 | *Sus scrofa* | UNIPROTKB\|F1RUT7 |
| comp573_c0_seq3 | 2925 | RNA-binding protein 4B | 129 | 371 | 0.012 | 1.752 | *Sus scrofa* | UNIPROTKB\|F1RUT7 |
| comp573_c0_seq4 | 2601 | RNA-binding protein 4B | 129 | 371 | 0.007 | 1.118 | *Sus scrofa* | UNIPROTKB\|F1RUT7 |
| comp24954_c0_seq1 | 3152 | roundabout | 362 | 2737 | 0.05 | 1.021 | *Drosophila melanogaster* | FB\|FBgn0005631 |
| comp24954_c0_seq2 | 3134 | roundabout | 362 | 2764 | 0.046 | 1.53 | *Drosophila melanogaster* | FB\|FBgn0005631 |
| comp24954_c0_seq3 | 2854 | roundabout | 46 | 2439 | 0.336 | 0.226 | *Drosophila melanogaster* | FB\|FBgn0005631 |
| comp24954_c0_seq4 | 2836 | roundabout | 46 | 2466 | 0.077 | 0.531 | *Drosophila melanogaster* | FB\|FBgn0005631 |
| comp26721_c0_seq1 | 7012 | roundabout | 442 | 2991 | 0.018 | 1.442 | *Drosophila melanogaster* | FB\|FBgn0005631 |
| comp26721_c0_seq2 | 5448 | roundabout | 3 | 1445 | 0.060 | 2.015 | *Drosophila melanogaster* | FB\|FBgn0005631 |
| comp45935_c0_seq1 | 1373 | roundabout | 1362 | 130 | 0.214 | 0.266 | *Drosophila melanogaster* | FB\|FBgn0005631 |
| comp60778_c0_seq1 | 488 | roundabout | 2 | 394 | 0.116 | 0.449 | *Drosophila melanogaster* | FB\|FBgn0005631 |
| comp67560_c0_seq1 | 506 | roundabout | 340 | 486 | 0.636 | 0.079 | *Drosophila melanogaster* | FB\|FBgn0005631 |
| comp45706_c0_seq1 | 482 | Serotonin receptor 1A | 3 | 386 | 0.687 | 0.073 | *Drosophila melanogaster* | FB\|FBgn0004168 |
| comp90243_c0_seq1 | 327 | Serotonin receptor 1A | 325 | 86 | 0.089 | 2.289 | *Drosophila melanogaster* | FB\|FBgn0004168 |
| comp96775_c0_seq1 | 235 | Shaker | 234 | 1 | 0.083 | 0.462 | *Drosophila melanogaster* | FB\|FBgn0003380 |
| comp19976_c0_seq1 | 1351 | slowpoke | 1053 | 910 | 0.808 | 0.096 | *Drosophila melanogaster* | FB\|FBgn0003429 |
| comp29144_c0_seq1 | 219 | slowpoke | 218 | 87 | 0.089 | 1.655 | *Drosophila melanogaster* | FB\|FBgn0003429 |
| comp62466_c0_seq1 | 411 | slowpoke | 275 | 409 | 0.125 | 0.301 | *Drosophila melanogaster* | FB\|FBgn0003429 |
| comp4789_c0_seq1 | 2620 | timeless | 2545 | 1181 | 0.16 | 0.243 | *Drosophila melanogaster* | FB\|FBgn0014396 |
| comp4789_c0_seq2 | 2526 | timeless | 2451 | 1087 | 0.322 | 0.169 | *Drosophila melanogaster* | FB\|FBgn0014396 |
| comp6413_c0_seq1 | 2451 | timeless | 1551 | 196 | 0.489 | 0.13 | *Drosophila melanogaster* | FB\|FBgn0014396 |
| **comp6413_c0_seq2 (-)** | 1966 | timeless | 1551 | 196 | 0.997 | 0.019 | *Drosophila melanogaster* | FB\|FBgn0014396 |
| comp39652_c0_seq1 | 1137 | TIMELESS (Uncharacterized protein) | 1135 | 458 | 0.042 | 0.959 | *Sus scrofa* | UNIPROTKB\|F1SLB6 |
| comp67299_c0_seq1 | 309 | timeout | 42 | 260 | 0.083 | 0.462 | *Drosophila melanogaster* | FB\|FBgn0038118 |
| comp7837_c0_seq1 | 1531 | vrille | 470 | 832 | 0.012 | 0.945 | *Drosophila melanogaster* | FB\|FBgn0016076 |

**S1 Text. Table D. List of representative transcripts that showed the highest levels of abundance during photophase and scotophase.** Transcripts were ordered by rank, together with the information related to their annotation (symbol of the hit, species, Go function and access number of the hit). PPDE (Posterior Probability of Differential Expression) represents the posterior probability of differential expression. post FC (L/D) represents the fold change of transcripts abundance between photophase/scotophase.

| **Rank** | **Transcript** | **PPDE** | **Post FC (L/D)** | **Symbol** | **Species** | **GO Function** | **Access number** |
| --- | --- | --- | --- | --- | --- | --- | --- |
| **PHOTOPHASE** | | | |  |  |  |  |
| 1 | comp188_c2_seq4 | 1.000000000 | 6.48E+03 | --- | --- | --- | no hit |
| 2 | comp2506_c0_seq2 | 1.000000000 | 4.55E+03 | CG42327 | *Drosophila melanogaster* | protein tyrosine phosphatase activity | FB\|FBgn0259227 |
| 3 | comp415_c0_seq7 | 1.000000000 | 3.69E+03 | --- | *Sus scrofa* | tight junction evidence | UNIPROTKB\|F1SMP5 |
| 4 | comp968_c1_seq4 | 1.000000000 | 2.72E+03 | capulet | *Drosophila melanogaster* | adenylate cyclase binding evidence | FB\|FBgn0261458 |
| 5 | comp1479_c0_seq10 | 1.000000000 | 2.15E+03 | --- | --- | --- | no hit |
| 6 | comp3626_c0_seq8 | 0.999999999 | 1.32E+03 | --- | --- | --- | no hit |
| **SCOTOPHASE** | | | |  |  |  |  |
| 1 | comp310_c0_seq2 | 1.000000000 | 1.20E-04 | --- | --- | --- | no hit |
| 2 | comp1372_c1_seq3 | 1.000000000 | 1.99E-04 | bent | *Drosophila melanogaster* | structural constituent of cytoskeleton | FB\|FBgn0005666 |
| 3 | comp439_c0_seq2 | 1.000000000 | 2.22E-04 | --- | --- | --- | no hit |
| 4 | comp259_c0_seq13 | 1.000000000 | 2.31E-04 | mhc | *Drosophila melanogaster* | striated muscle contraction | FB\|FBgn0086783 |
| 5 | comp1307_c0_seq2 | 1.000000000 | 2.86E-04 | MSF3 | *Drosophila melanogaster* | high affinity inorganic phosphate | FB\|FBgn0031307 |
| 6 | comp1154_c0_seq2 | 1.000000000 | 2.98E-04 | --- | --- | --- | no hit |
| 7 | comp802_c0_seq2 | 1.000000000 | 3.30E-04 | Beta-carotene dioxygenase 1 | *Gallus gallus* | retinoid metabolic process | UNIPROTKB\|Q9I993 |
| 8 | comp712_c4_seq4 | 1.000000000 | 3.31E-04 | --- | --- | --- | no hit |
| 9 | comp1364_c1_seq2 | 0.999999999 | 3.95E-04 | cdk16 | *Danio rerio* | transferring phosphorus-containing groups | ZFIN\|ZDB-GENE-030131-2939 |
| 10 | comp676_c0_seq2 | 0.999999999 | 4.06E-04 | FK506-binding protein 2 | *Drosophila melanogaster* | FK506 binding evidence | FB\|FBgn0013954 |
| 11 | comp1372_c1_seq1 | 0.999999998 | 4.83E-04 | bent | *Drosophila melanogaster* | structural constituent of cytoskeleton | FB\|FBgn0005666 |
| 12 | comp2261_c0_seq2 | 0.999999998 | 5.10E-04 | --- | --- | --- | no hit |
| 13 | comp157_c0_seq4 | 0.999999998 | 5.23E-04 | Rrh | *Mus musculus* | retinal pigment epithelium rhodopsin homolog | MGI\|MGI:1097709 |
| 14 | comp2004_c0_seq4 | 0.999999997 | 5.60E-04 | CG6296 | *Drosophila melanogaster* | triglyceride lipase activity | FB\|FBgn0039470 |
| 15 | comp259_c0_seq19 | 0.999999997 | 5.62E-04 | myosin heavy chain | *Drosophila melanogaster* | striated muscle contraction | FB\|FBgn0086783 |

**S1 Text. Table E. Blastx of the contig annotated to *cryptochrome***

| **Genes** | **Species** | **Phylum - Class -Order** | **Definition** | **Identity** | **Gaps** | **Accession** |
| --- | --- | --- | --- | --- | --- | --- |
| *cryptochrome* | *Euphausia superba* | Arthropoda - Malacostraca - Euphasiacea | cryptochrome | 441/539 | 3/539 | CAQ86665 |
|  | *Eurydice pulchra* | Arthropoda - Malacostraca - Isopoda | cryptochrome 2 | 421/541 | 4/541 | AGV28717 |
|  | *Talitrus saltator* | Arthropoda - Malacostraca - Anphipoda | cryptochrome 2 | 396/499 | 0/499 | AFV96168 |

**S1 Text. Table F. Conserved domains of canonical clock genes in *Nephrops norvegicus***

| **Protein** | **Conserved domain** | **Position (aa)** | **Aminoacid sequence** |
| --- | --- | --- | --- |
| PERIOD | PAS domain | 229-296 | AAFLKSFKSTRGFTVAISVQDGTVLQVSPAITDVLGFPKDMLIGQSFIDFVYPKDSINLSSKIIHGLN |
|  | PAS domain | 373-442 | ESIYTVPEETPAMGSFSIRHSASCNFSEYDPEAIPYLGHLPQDLTGNSVFDCYHXEDLPLLKAVYEGMVR |
|  | PAC motif | 450-493 | SKPYRFRTFNGSYVTLQTEWLCFVNPWTKRIDSIIGQHRVLKGP |
| BMAL1 | bHLH | 41-74 | SEIEKRRRDKMNTYIMELSSIIPVCTSRKLDKLT |
